# Inverted U-shaped relationship between serum 25-hydroxyvitamin D concentrations and *Toxoplasma gondii* infection: a cross-sectional study

**DOI:** 10.3389/fpubh.2024.1420932

**Published:** 2024-08-29

**Authors:** Lihua Huang, Xiaoyan Luo, Liuliu He, Xiaoyan You, Xiaobo Chen

**Affiliations:** ^1^Department of Clinical Laboratory, The Second Affiliated Hospital of Gannan Medical University, Ganzhou, China; ^2^Department of Interventional Radiology, The Second Affiliated Hospital of Gannan Medical University, Ganzhou, China; ^3^Department of Rehabilitation Medicine, The Second Affiliated Hospital of Gannan Medical University, Ganzhou, China

**Keywords:** serum 25-hydroxyvitamin D, parasite, toxoplasmosis, non-linear association, cross-sectional study

## Abstract

**Background:**

*Toxoplasma gondii* (*T. gondii*) is a widespread zoonotic parasite transmitted through contaminated food or water. It poses a significant public health threat, especially to pregnant women and immunocompromised individuals. 25-Hydroxyvitamin D [25(OH)D] plays a critical role in regulating both innate and adaptive immune responses, particularly in its anti-infective capacity. However, the relationship between serum 25(OH)D concentrations and *T. gondii* infection remains uncertain.

**Methods:**

We analyzed the data from the National Health and Nutrition Examination Survey (NHANES) spanning 2009–2014 to explore the association between serum 25(OH)D concentrations and *T. gondii* infection. Extensive demographic, comorbidity, and dietary data were collected. The status of *T. gondii* infection was determined using serum anti-IgG antibodies. Serum 25(OH)D levels were measured using ultra-high performance liquid chromatography–tandem mass spectrometry (UHPLC–MS/MS). In addition, weighted logistic regression and restricted cubic spline analyses were performed.

**Results:**

Our analysis included 10,157 participants (mean [SE] age, 45.38 [0.39] years; 49.73% female) who met the inclusion criteria. Serum 25(OH)D levels were categorized into quintiles, with the second quintile serving as the reference group. The final model, adjusted for age, sex, race, education level, poverty income ratio, body mass index, smoking status, hypertension, diabetes, chronic kidney disease, depression, physical activity, alcohol intake, seasonal testing, and dietary vitamin D, revealed the following adjusted odds ratios (ORs) for the quintiles: 0.75 (95% confidence interval [CI]: 0.60–0.93) for the first, 0.87 (95% CI: 0.69–1.10) for the third, 0.75 (95% CI: 0.58–0.95) for the fourth, and 0.66 (95% CI: 0.49–0.91) for the fifth. Additionally, a restricted cubic spline analysis revealed an inverted U-shaped relationship between serum 25(OH)D and *T. gondii* infection, with an inflection point at approximately 51.29 nmol/L. Odds ratios to the left and right of the inflection point were 1.17 (95% CI: 1.03–1.32) and 0.94 (95% CI, 0.90–0.98) per 10 nmol/L, respectively.

**Conclusion:**

Our study uncovers an inverted U-shaped relationship between serum 25(OH)D concentrations and *T. gondii* infection, with an inflection point around 51.29 nmol/L.

## Introduction

*Toxoplasma gondii* (*T. gondii*) is a prevalent zoonotic parasite that is commonly transmitted through the consumption of food or water contaminated with oocysts excreted by cats or by ingesting undercooked meat products (1). This parasite exists in three primary forms, namely, oocysts, tachyzoites, and bradyzoites. Oocysts are exclusively produced within feline hosts, whereas tachyzoites are found in both humans and other intermediary hosts. The ingestion of tachyzoites by humans or intermediary hosts is followed by their rapid multiplication within the intestinal epithelium and dissemination throughout the body via the lymphatic system (2). Infection with *T. gondii* can lead to opportunistic infections in immunocompromised individuals, possibly causing severe congenital defects or miscarriages (3). The global prevalence of *T. gondii* infections is significant, affecting approximately 30 to 50% of the world’s population, with an estimated prevalence of 10.4% among individuals aged 6 years and older in the United States (4). These infections not only have a substantial impact on public health but also impose a considerable economic burden. They rank as the third leading cause of foodborne infections requiring hospitalization in the United States (5), indicating that controlling the transmission of *T. gondii* is an urgent priority. The body’s innate immune response restrains parasite growth and fosters the development of adaptive immunity, which is crucial for establishing long-term resistance to infection (6).

25-hydroxyvitamin D [25(OH)D], the primary storage form of vitamin D, is a fat-soluble sterol. In addition to regulating calcium, it has been known to modulate the immune system’s responses to parasites. Research has demonstrated that 25(OH)D influences the defense against pathogens by balancing T helper lymphocyte subpopulations, with a particular emphasis on Th2 cells, which are involved in anti-inflammation and combatting helminth infections (7). In addition, vitamin D exerts its anti-plasmodial effects by inducing the production of lactoferrin and chelicidin-related antimicrobial peptides, while also regulating the levels of reactive oxygen species and inhibiting phospholipid biosynthesis, among other mechanisms (8). Furthermore, supplementation with 25(OH)D during the muscular stage of trichinellosis has demonstrated favorable inhibitory effects (9). However, despite the similarities in intracellular parasitism between *T. gondii* and the aforementioned parasites, the exact association between 25(OH)D and *T. gondii* infection remains unclear. Intriguingly, an *in vitro* experiment demonstrated that mice infected with *T. gondii* had a higher mortality rate than the other groups following treatment with 1,25(OH)2D3, the catalyzed form of 25(OH)D3 in the kidney (10). However, their levels of inflammatory cytokines (interferon [IFN]-γ and interleukin [IL]-12, p40) were significantly reduced, and a further *in vitro* cellular assay revealed that intracellular 1,25(OH)2D3 treatment led to a dose-dependent reduction in parasite numbers following *T. gondii* infection of mouse intestinal epithelial cells (11). However, the relationship between serum 25(OH)D levels and *T. gondii* infection has been less well-studied and demonstrated controversies in population-based observational studies (12–14). Therefore, we investigated the relationship between serum 25(OH)D levels and *T. gondii* infection in greater depth using a comprehensive dataset obtained from the National Health and Nutrition Examination Survey (NHANES).

## Methods

### Data source and study design

**Figure 1:**
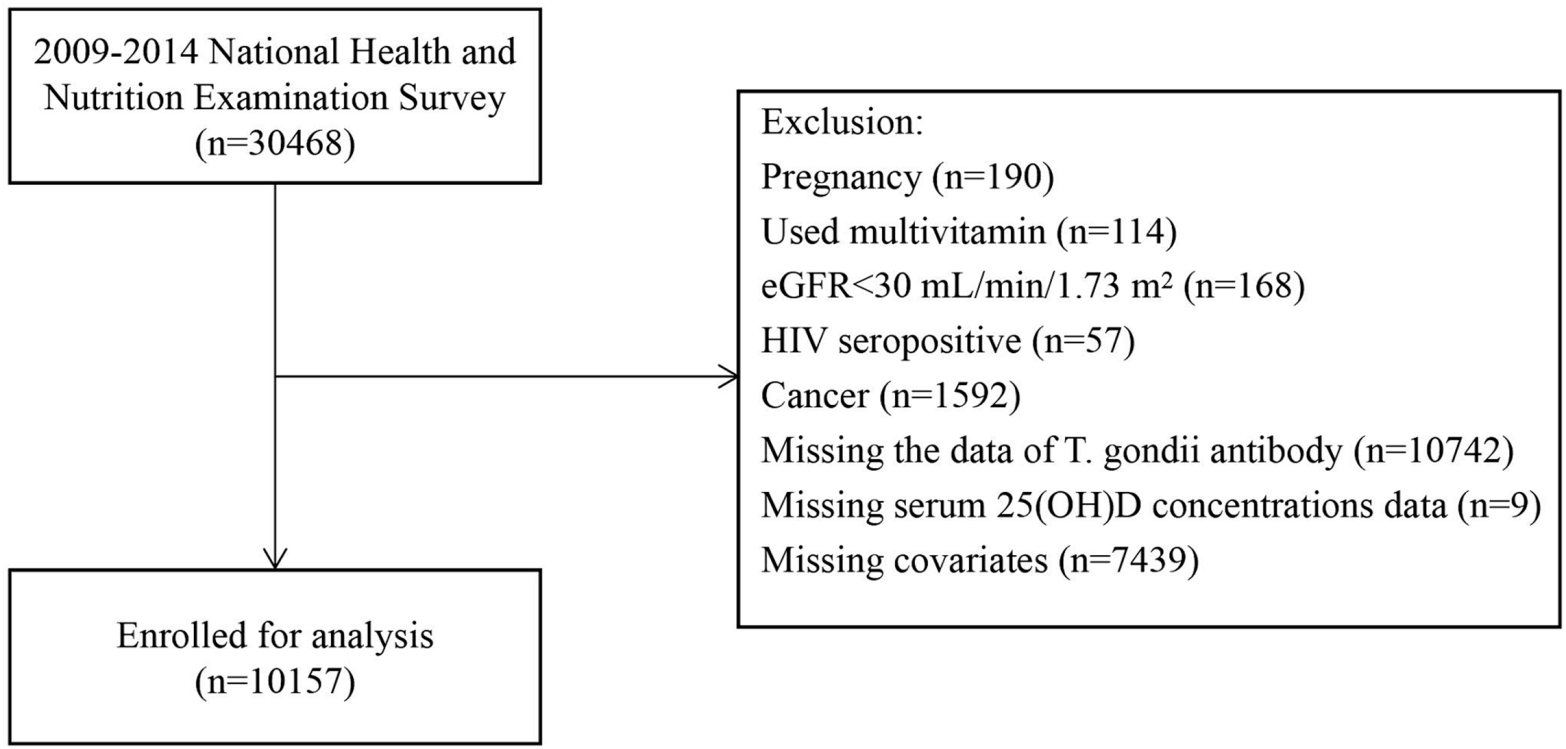
Study flowchart.

NHANES is conducted by the National Center for Health Statistics (NCHS), a division of the Centers for Disease Control and Prevention (CDC). This national survey assesses the health status of the entire U.S. population. We used a multistage probability sampling approach to collect the annually over 2 years. We sourced the data related to non-institutionalized civilians from NHANES spanning from 2009 to 2014. We collated demographic information, details on diseases, laboratory results, and questionnaire data related to disease classification. The purity of our data concerning serum 25(OH)D levels was ensured by employing strict criteria, drawing upon relevant literature and clinical expertise. Ultimately, we excluded individuals who reported pregnancy at baseline (*n* = 190), those who had used multivitamins within the past 1 month (*n* = 114), those with an estimated glomerular filtration rate (eGFR) of less than 30 mL/min/1.73 m^2^ (*n* = 168), those who were tested positive for human immunodeficiency virus (HIV; *n* = 57), those who self-reported cancer (*n* = 1,592), participants with missing *T. gondii* test data (*n* = 10,742), individuals with missing serum 25(OH)D levels data (*n* = 9), and those lacking data on other covariates (*n* = 7,439). We excluded individuals who had used multivitamins due to the potential interference of exogenous vitamin D with the relationship between serum 25(OH)D and *T. gondii* infection. Additionally, those with HIV, cancer, or an eGFR of less than 30 mL/min/1.73 m^2^ were excluded because their immunocompromised status makes them more susceptible to *T. gondii* infection, which could significantly influence the conclusions. Our final analysis included 10,157 participants, as illustrated in Figure 1. The Institutional Review Board (IRB) of the CDC’s National Center for Health Statistics (NCHS) authorized the NHANES website^1^ to provide public access to study data. To ensure population representativeness, our statistical analyses followed the NHANES Analytic Guidelines, employing the provided check sample weights.^2^ The NHANES study obtained ethical approval from the NCHS IRB under Protocols 98–12, 2005–06, 2011–17, and 2018–01, and all participants provided informed consent.

### Definition of *Toxoplasma Gondii* infection

The *T. gondii* anti-IgG antibodies were measured using the *Toxoplasma* IgG EIA assay (Bio-Rad; Redmond, WA, United States). A value equal to or greater than 33 IU/mL was considered positive, whereas a value less than 27 IU/mL was deemed negative. Results falling between 27 and 33 IU/mL were considered equivocal. Equivocal results were confirmed using at least two repeated tests and were subsequently categorized as negative. Each plate included quality control samples to ensure accuracy and consistency. The reported results were presented as either index values or IU/mL, traceable back to the World Health Organization (WHO) anti-*Toxoplasma* serum, 3rd International Standard Preparation from 1994. Before conducting the study, the performance of the assay was evaluated against the CDC *Toxoplasma* using the immunofluorescence assay and the Sabin–Feldman dye test, achieving a notable 100% sensitivity and specificity. Serological test results served as valuable presumptive evidence for *T. gondii* infection.

### Measurement of serum 25(OH)D concentrations

For the NHANES 2009–2014 study, blood samples collected during the MEC examination were initially processed and stored at −30°C. Subsequently, these samples were transported to the CDC Environmental Health Laboratory in Atlanta, Georgia, for comprehensive analysis. A standardized technique using ultra-high performance liquid chromatography–tandem mass spectrometry (UHPLC–MS/MS) was employed to determine the concentrations of both serum 25-hydroxyvitamin D3 [25(OH)D3] and 25-hydroxyvitamin D2 [25(OH)D2]. The overall serum 25(OH)D concentrations were determined by summing the concentrations of both 25(OH)D3 and 25(OH)D2. The study groups were categorized into five distinct groups based on their serum 25(OH)D concentrations, each representing a quintile. The specific cut-off values for these groupings were as follows: quintile 1 (<40.65 nmol/L), quintile 2 (40.66–55.25 nmol/L), quintile 3 (55.26–67.66 nmol/L), quintile 4 (67.67–83.95 nmol/L), and quintile 5 (>83.96 nmol/L).

### Covariates

We collected a variety of covariates, encompassing age, gender, race/ethnicity (categorized as Mexican American, other Hispanic, non-Hispanic White, non-Hispanic Black, or other races including multi-racial), body mass index (BMI), education level (categorized as <9 years, 9–13 years, ≥13 years), poverty-to-income ratio (calculated by dividing the household income by the poverty threshold and adjusted for household size, year, and state), smoking status (categorized as current, former, and never smoker), diabetes (self-reported physician-diagnosed, use of glucose-lowering medications, or a glycosylated hemoglobin level ≥ 6.5%), hypertension (defined as systolic blood pressure ≥ 140 mm Hg, diastolic blood pressure ≥ 90 mm Hg, self-reported physician-diagnosed hypertension, or use of antihypertensive medication), physical activity (categorized as active or inactive), and alcohol consumption (categorized as none, moderate: 0.1–27.9 g/day in men and 0.1 to 13.9 g/day in women, or heavy: ≥28 g/day in men and ≥ 14 g/day in women) (15). The season of testing was classified as May 1 through October 31 or November 1 through April 30. The total vitamin D intake was determined by assessing the 24-h dietary consumption. According to the Kidney Disease Improving Global Outcomes guidelines, any chronic kidney disease (CKD) was defined as an eGFR less than 60 mL/min per 1.73 m^2^ or an albumin-to-creatinine ratio (ACR) equal to or exceeding 30 mg/g (16). The depressive status was assessed using the nine-item Patient Health Questionnaire (PHQ-9) during the initial evaluation, with a score of 10 or higher indicating the presence of depressive symptoms (17). In addition, depression (yes and no) was included as a confounding factor in our analysis, as the previous literature has demonstrated an association with *T. gondii* infection (18).

### Statistical analysis

Weighted continuous data are reported as mean (standard error [SE]), and group differences were assessed via analysis of variance (ANOVA) or the Kruskal–Wallis test, contingent on the distribution of continuous variables. Categorical data are expressed in numbers (*n*) and weighted percentages (%), and group distinctions were scrutinized employing chi-square tests. The association between serum 25(OH)D concentrations and *T. gondii* infection was assessed using the weighted logistic regression models. The quintile 2 was used as a reference group, Model I was adjusted for age (continuous, in years) and gender (male or female). In Model II, we further adjusted for race/ethnicity (categorized as Mexican American, other Hispanic, non-Hispanic White, non-Hispanic Black, or other races including multi-racial), education level (<9 years, 9–13 years, or ≥ 13 years), poverty-to-income ratio (continuous), BMI (continuous), smoking status (never smoker, former smoker, and current smoker), and comorbidities (diabetes mellitus, hypertension, kidney disease, and depression). In Model III, additional adjustments were made for physical activity (inactive or active), alcohol intake (none, moderate, or heavy), season of testing (May 1 through October 31 or November 1 through April 30), and dietary vitamin D intake (continuous).

Furthermore, we used a two-piecewise linear regression model with a smoothing curve to investigate the non-linear relationship between serum 25(OH)D concentrations and *T. gondii* infection. Likelihood ratio tests were conducted to compare the goodness-of-fit between one-line linear regression and two-piecewise linear regression models. The threshold value was identified as the point with the highest likelihood among all potential values.

Subgroup analyses were conducted based on several factors, including age (<60 or ≥ 60 years), poverty income ratio (<1, 1–3, ≥3), race-ethnicity (Mexican American, other Hispanic, non-Hispanic White, non-Hispanic Black, or other races including multi-racial), BMI (<24.9, 25.0–29.9, or ≥ 30 kg/m^2^), smoking status (never smoker, former smoker, or current smoker), diabetes (yes or no), hypertension (yes or no), CKD (yes or no), depression (yes or no), season of testing (May 1 through October 31 or November 1 through April 30), physical activity (inactive or active), and alcohol intake (none, moderate, or heavy). Interaction effects were assessed using *p*-values for the product terms between anti-*T. gondii* antibody status (negative or positive) and stratified factors.

We conducted sensitivity analyses to bolster the robustness of our findings. Missing data were addressed through multiple imputations employing five replications of the Markov chain Monte Carlo method.

All statistical analyses were performed using the R software (version 4.3.1). A two-sided *p*-value <0.05 was considered significant.

## Results

### Study participants and baseline characteristics

Baseline characteristics of the subjects were categorized into quintiles based on serum 25(OH)D concentrations (Table 1). A total of 10,157 patients afflicted with *T. gondii* infection who satisfied the inclusion criteria were identified (Figure 1). As indicated in Table 1, individuals with higher 25(OH)D concentrations were more likely to be female, non-Hispanic White, older, with a low BMI. They exhibited a lower current smoking rate, a higher prevalence of hypertension and CKD, a lower prevalence of diabetes and depression, increased physical activity, higher alcohol intake, and were more likely to be tested between May 1 and October 31. In addition, they had lower rates of *T. gondii* infection. When grouped by *T. gondii* infection status (serum IgG positive), those infected with *T. gondii* were older, more likely to be male, Other Hispanic, had lower poverty income ratio, lower education level, higher BMI, a higher prevalence of chronic diseases (hypertension, diabetes, CKD), engaged in less physical activity, and consumed less alcohol (Supplementary Table S1).

**Table 1:** Baseline characteristics of the study participant.

variable		Serum 25(OH)D concentrations (nmol/L)					
		<40.65	40.66–55.25	55.26–67.66	67.67–83.95	>83.96	
	Total (*n* = 10,157)	Quintile1(*n* = 2037)	Quintile2 (*n* = 2030)	Quintile3 (*n* = 2027)	Quintile4 (*n* = 2033)	Quintile5 (*n* = 2030)	*P*-value
Age, years	45.38 (0.39)	41.64 (0.41)	42.20 (0.56)	43.70 (0.50)	45.52 (0.56)	51.08 (0.66)	< 0.001
Sex, n (%)							< 0.001
Female	5,060 (49.73)	1,067 (52.36)	928 (45.46)	913 (43.97)	945 (45.45)	1,207 (59.82)	
Male	5,097 (50.27)	970 (47.64)	1,102 (54.54)	1,114 (56.03)	1,088 (54.55)	823 (40.18)	
Race-ethnicity, n (%)							< 0.001
Mexican American	1,514 (8.58)	371 (15.09)	432 (14.19)	372 (10.45)	233 (5.54)	106 (2.07)	
Non-Hispanic Black	1943 (10.32)	825 (31.71)	418 (12.66)	267 (6.91)	228 (4.69)	205 (3.97)	
Non-Hispanic White	4,614 (68.78)	394 (34.60)	670 (55.50)	952 (70.31)	1,198 (80.04)	1,400 (86.78)	
Other Hispanic	984 (5.51)	177 (6.88)	256 (8.76)	234 (6.62)	177 (4.23)	140 (2.68)	
Other Race - Including Multi-Racial	1,102 (6.81)	270 (11.71)	254 (8.89)	202 (5.71)	197 (5.50)	179 (4.50)	
Poverty Income Ratio	2.95 (0.06)	2.37 (0.06)	2.68 (0.08)	2.83 (0.07)	3.21 (0.07)	3.35 (0.06)	< 0.001
Education Level, n (%)							< 0.001
Low (<9 years)	836 (4.35)	146 (4.54)	212 (6.25)	223 (5.74)	151 (3.53)	104 (2.56)	
Medium (9–13 years)	3,798 (33.38)	892 (42.25)	767 (34.82)	744 (33.01)	700 (30.57)	695 (30.00)	
High (≥13 years)	5,523 (62.27)	999 (53.21)	1,051 (58.93)	1,060 (61.25)	1,182 (65.90)	1,231 (67.44)	
BMI, kg/m^2^	28.97 (0.12)	31.01 (0.22)	30.10 (0.24)	29.33 (0.21)	28.35 (0.21)	27.24 (0.20)	< 0.001
Smoking status, n (%)							< 0.001
Former Smoker	2,305 (23.17)	332 (15.92)	406 (18.31)	477 (23.85)	531 (26.52)	559 (27.27)	
Never Smoker	5,678 (56.62)	1,152 (56.54)	1,179 (59.10)	1,127 (56.27)	1,099 (55.05)	1,121 (56.65)	
Current Smoker	2,174 (20.21)	553 (27.54)	445 (22.59)	423 (19.89)	403 (18.43)	350 (16.08)	
Hypertension, n (%)							0.004
No	6,233 (65.05)	1,257 (63.19)	1,297 (67.03)	1,323 (67.97)	1,263 (66.41)	1,093 (61.15)	
Yes	3,924 (34.95)	780 (36.81)	733 (32.97)	704 (32.03)	770 (33.59)	937 (38.85)	
Diabetes, n (%)							< 0.001
No	8,708 (89.49)	1716 (86.20)	1715 (87.58)	1739 (89.47)	1781 (91.43)	1757 (90.99)	
Yes	1,449 (10.51)	321 (13.80)	315 (12.42)	288 (10.53)	252 (8.57)	273 (9.01)	
CKD							< 0.001
No	8,617 (87.66)	1727 (86.26)	1750 (89.33)	1772 (90.00)	1749 (88.62)	1,619 (84.52)	
Yes	1,540 (12.34)	310 (13.74)	280 (10.67)	255 (10.00)	284 (11.38)	411 (15.48)	
Depression, n (%)							0.01
No	9,194 (91.83)	1814 (89.95)	1826 (90.87)	1844 (91.16)	1850 (92.21)	1860 (93.82)	
Yes	963 (8.17)	223 (10.05)	204 (9.13)	183 (8.84)	183 (7.79)	170 (6.18)	
Physical Activity, n (%)							< 0.001
Inactive	4,996 (44.14)	1,182 (56.68)	1,047 (49.48)	1,001 (45.68)	912 (40.08)	854 (35.45)	
Active	5,161 (55.86)	855 (43.32)	983 (50.52)	1,026 (54.32)	1,121 (59.92)	1,176 (64.55)	
Alcohol Intake, n (%)							< 0.001
None	7,711 (72.25)	1,580 (75.09)	1,582 (77.14)	1,577 (74.52)	1,509 (70.19)	1,463 (67.18)	
Moderate	711 (7.23)	99 (5.05)	137 (6.20)	150 (8.13)	165 (8.12)	160 (7.70)	
Heavy	1735 (20.53)	358 (19.86)	311 (16.66)	300 (17.36)	359 (21.70)	407 (25.12)	
Seasonal Testing, n (%)							< 0.001
May 1 through October 31	5,267 (56.11)	708 (34.23)	959 (48.93)	1,099 (57.12)	1,213 (62.32)	1,288 (67.62)	
November 1 through April 30	4,890 (43.89)	1,329 (65.77)	1,071 (51.07)	928 (42.88)	820 (37.68)	742 (32.38)	
Dietary vitamin D, (mg)	4.81 (0.08)	3.46 (0.11)	4.48 (0.10)	4.89 (0.19)	5.15 (0.16)	5.45 (0.25)	< 0.001
*Toxoplasma gondii* IgG antibody, n (%)							0.001
Negative	8,535 (87.24)	1742 (87.47)	1,670 (84.13)	1,667 (86.13)	1733 (88.47)	1723 (89.06)	
Positive	1,622 (12.76)	295 (12.53)	360 (15.87)	360 (13.87)	300 (11.53)	307 (10.94)	

Data are represented as the weighted proportion (%) or mean ± SE. BMI, body mass index; CVD, cardiovascular disease.

### Relationships of 25(OH)D concentration with *Toxoplasma Gondii* infection

We formulated three regression models to explore the independent association of serum 25(OH)D levels with *T. gondii* infection. Following full adjustment for covariates in model III, a comparison to quintile 2 (40.66–55.25 nmol/L) revealed that the adjusted odds ratios (ORs) for quintile 1 (<40.65 nmol/L), quintile 3 (55.26–67.66 nmoL/L), quintile 4 (67.67–83.95 nmoL/L), and quintile 4 (>83.96 nmoL/L) were 0.75 (95% CI: 0.60–0.93), 0.87 (95% CI: 0.69–1.10), 0.75 (95% CI: 0.58–0.95), and 0.66 (95% CI: 0.49–0.91), respectively, after all covariate adjustment in model III (Table 2).

**Table 2:** ORs and 95% CIs of *Toxoplasma gondii* infection according to serum 25(OH)D levels.

Variable	Total	*T. gondii*-seropositive	Non-adjusted model		Model I		Model II		Model III	*P*-value
	n	n (%)	HR (95% CI)		HR (95% CI)		HR (95% CI)		HR (95% CI)	
Serum 25(OH)D levels (nmol/L)										
Quintile 1 (<40.65)	2037	295 (12.53)	0.76 (0.61–0.94)	0.012	0.78 (0.63–0.96)	0.021	0.73 (0.59–0.91)	0.006	0.75 (0.60–0.93)	0.012
Quintile 2 (40.46–55.25)	2030	360 (15.87)	Ref.		Ref.		Ref.		Ref.	
Quintile 3 (55.26–67.66)	2027	360 (13.87)	0.85 (0.69–1.06)	0.15	0.80 (0.64–0.99)	0.044	0.87 (0.69–1.10)	0.236	0.87 (0.69–1.10)	0.228
Quintile 4 (67.67–83.95)	2033	300 (11.53)	0.69 (0.55–0.86)	0.002	0.61 (0.49–0.77)	<0.001	0.74 (0.58–0.94)	0.017	0.75 (0.58–0.95)	0.021
Quintile 5 (>83.96)	2030	307 (10.94)	0.65 (0.49–0.86)	0.004	0.50 (0.38–0.67)	<0.001	0.65 (0.48–0.89)	0.01	0.66 (0.49–0.91)	0.012
P for trend				0.006		<0.001		0.050		0.054

Model I: adjusted for age + sex; Model II: adjusted for Model I + race + education + poverty income ratio + BMI + smoking status + hypertension + diabetes + CKD + depression; Model III: adjusted for Model II + physical activity + alcohol intake + seasonal testing + dietary vitamin D.BMI, body mass index; *T. gondii*, *Toxoplasma gondii*; CKD, chronic kidney disease.

### Non-linear relationship between 25(OH)D concentration and *Toxoplasma Gondii* infection

**Figure 2:**
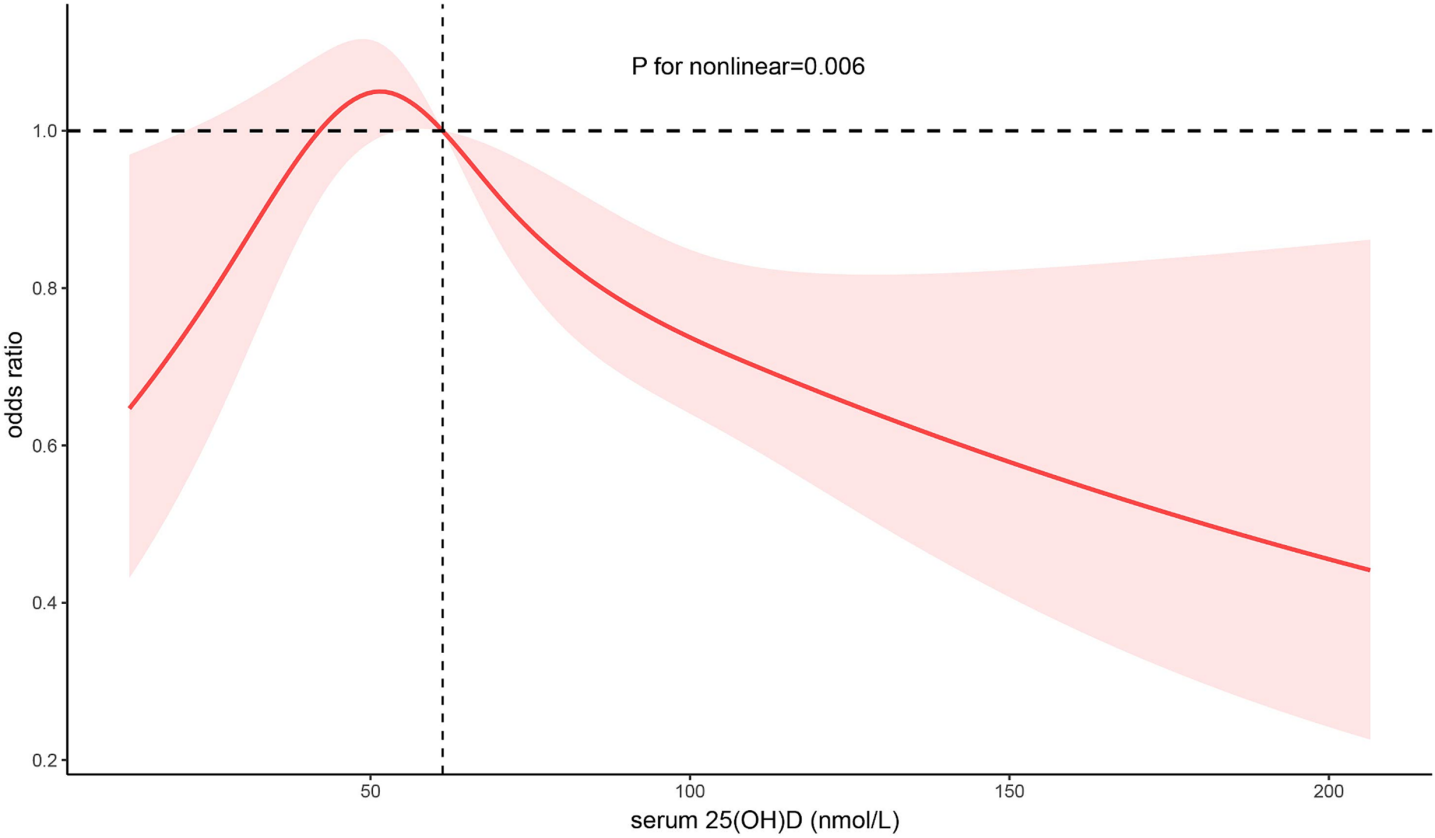
Multivariable adjusted spline curves for associations of the serum 25-hydroxyvitamin D with *Toxoplasma gondii* infection Adjusted for age, sex, race, education, poverty income ratio, BMI, smoking status, hypertension, diabetes, CKD, depression, physical activity, alcohol intake, seasonal testing, dietary vitamin D. BMI, body mass index.

We applied a multivariate logistic regression model and a smoothed curve fit and identified an inverted U-shaped association between 25(OH)D concentration and *T. gondii* infection across the entire study population (Figure 2). We used a pairwise multivariate logistic regression model to derive two distinct slopes. We used a two-segment model to describe the relationship between 25(OH)D concentration and *T. gondii* infection, where the log-likelihood ratio test yielded a *p*-value of 0.002 (Table 3). The OR to the left of the inflection point at 51.29 nmol/L was 1.17 (95% CI: 1.03–1.32), per 10 nmol/L, whereas it was 0.94 (95% CI: 0.90–0.98), per 10 nmol/L, right of the inflection point.

**Table 3:** Threshold effect analysis of serum 25-hydroxyvitamin D concentrations and *Toxoplasma gondii* infection.

Threshold of driving pressure	OR	95% CI	*P*-value
Serum 25(OH)D < 51.29 (per 10 nmol/L)	1.17	1.03–1.32	0.016
Serum 25(OH)D ≥ 51.29 (per 10 nmol/L)	0.94	0.90–0.98	0.007
Likelihood Ratio test			0.002

Adjusted for age + sex + race + education + poverty income ratio + BMI + smoking status + hypertension + diabetes + CKD + depression + physical activity + alcohol intake + seasonal testing + dietary vitamin D.

### Stratified and sensitivity analyses

Stratified analysis was conducted to detect no discernible signs of alterations in the non-linear relationship between 25(OH)D concentration and *T. gondii* infection concerning several factors. (Table 4). Furthermore, we detected no statistically significant interactions within any of the strata (P for interaction >0.05). Notably, our multiple imputation sensitivity analysis did not substantially affect the aforementioned findings (Supplementary Table S2).

**Table 4:** Associations between serum 25-hydroxyvitamin D concentrations with *Toxoplasma gondii* infection in various subgroups.

	Serum 25-hydroxyvitamin D concentrations (nmol/L)					
Character	Quintile 1 (<40.65)	Quintile 2 (40.46–55.25)	Quintile 3 (55.26–67.66)	Quintile 4 (67.67–83.95)	Quintile 5 (>83.96)	*p* for interaction
Age						0.150
<60	0.76 (0.58–0.98)	Ref.	0.84 (0.61–1.14)	0.77 (0.57–1.04)	0.57 (0.36–0.90)	
≥60	0.65 (0.41–1.03)	Ref.	0.96 (0.63–1.47)	0.73 (0.47–1.11)	0.77 (0.50–1.17)	
Sex						0.554
Male	0.76 (0.56–1.03)	Ref.	0.92 (0.64–1.31)	0.67 (0.46–0.98)	0.64 (0.40–1.03)	
Female	0.72 (0.51–1.03)	Ref.	0.78 (0.53–1.13)	0.84 (0.59–1.18)	0.68 (0.45–1.02)	
Race-ethnicity						0.239
Non-Hispanic White	0.69 (0.46–1.06)	Ref.	0.78 (0.54–1.13)	0.73 (0.50–1.06)	0.64 (0.42–0.98)	
Non-Hispanic Black	0.59 (0.41–0.86)	Ref.	0.71 (0.38–1.35)	0.56 (0.29–1.11)	0.53 (0.30–0.95)	
Mexican American	0.86 (0.57–1.30)	Ref.	1.49 (1.02–2.17)	1.01 (0.67–1.54)	0.82 (0.37–1.79)	
Other Hispanic	0.89 (0.52–1.54)	Ref.	1.30 (0.83–2.05)	1.06 (0.61–1.85)	0.73 (0.37–1.42)	
Other Race	0.81 (0.40–1.61)	Ref.	0.41 (0.20–0.84)	0.42 (0.21–0.85)	0.47 (0.24–0.93)	
Education Level						0.550
Low (<9 years)	0.61 (0.33–1.12)	Ref.	1.19 (0.65–2.16)	0.60 (0.28–1.31)	1.01 (0.46–2.21)	
Medium (9–13 years)	0.81 (0.57–1.15)	Ref.	0.94 (0.65–1.35)	0.87 (0.59–1.27)	0.68 (0.47–1.00)	
High (≥13 years)	0.74 (0.54–1.00)	Ref.	0.76 (0.56–1.04)	0.70 (0.51–0.95)	0.60 (0.42–0.87)	
Poverty Income Ratio						0.616
<1	0.88 (0.58–1.32)	Ref.	0.74 (0.48–1.15)	0.79 (0.47–1.35)	0.76 (0.41–1.41)	
1.0–3.0	0.73 (0.49–1.09)	Ref.	1.01 (0.69–1.46)	0.90 (0.60–1.35)	0.78 (0.48–1.28)	
≥3	0.65 (0.39–1.06)	Ref.	0.78 (0.51–1.17)	0.59 (0.41–0.84)	0.51 (0.35–0.75)	
BMI- kg/m2						0.523
<24.9	0.90 (0.51–1.59)	Ref.	0.97 (0.55–1.71)	0.74 (0.45–1.21)	0.68 (0.40–1.16)	
25.0–29.9	0.79 (0.52–1.20)	Ref.	0.73 (0.51–1.06)	0.72 (0.47–1.08)	0.61 (0.38–0.97)	
≥30	0.67 (0.47–0.96)	Ref.	0.96 (0.66–1.40)	0.79 (0.54–1.16)	0.72 (0.47–1.11)	
Seasonal Testing						0.687
November 1 through April 30	0.87 (0.63–1.19)	Ref.	0.94 (0.66–1.36)	0.84 (0.58–1.22)	0.74 (0.46–1.19)	
May 1 through October 31	0.63 (0.43–0.94)	Ref.	0.79 (0.57–1.09)	0.66 (0.46–0.95)	0.60 (0.36–1.00)	
Smoking status						0.754
Former Smoker	0.74 (0.46–1.18)	Ref.	0.88 (0.56–1.38)	0.79 (0.49–1.28)	0.59 (0.38–0.91)	
Current Smoker	0.60 (0.36–0.99)	Ref.	0.87 (0.47–1.61)	0.50 (0.30–0.84)	0.68 (0.36–1.29)	
Never Smoker	0.81 (0.61–1.07)	Ref.	0.87 (0.63–1.22)	0.84 (0.56–1.25)	0.71 (0.49–1.05)	
Hypertension						0.499
No	0.85 (0.64–1.13)	Ref.	0.84 (0.63–1.12)	0.78 (0.56–1.10)	0.66 (0.44–0.98)	
Yes	0.62 (0.43–0.89)	Ref.	0.93 (0.69–1.25)	0.70 (0.48–1.01)	0.65 (0.46–0.92)	
DM						0.460
No	0.78 (0.61–1.01)	Ref.	0.88 (0.67–1.14)	0.72 (0.55–0.94)	0.65 (0.46–0.92)	
Yes	0.59 (0.37–0.94)	Ref.	0.82 (0.45–1.51)	0.96 (0.58–1.59)	0.75 (0.45–1.23)	
CKD						0.984
No	0.76 (0.59–0.99)	Ref.	0.85 (0.65–1.12)	0.73 (0.55–0.98)	0.65 (0.46–0.92)	
Yes	0.70 (0.41–1.20)	Ref.	1.00 (0.56–1.78)	0.84 (0.40–1.76)	0.74 (0.45–1.23)	
Depression						0.637
No	0.77 (0.60–0.97)	Ref.	0.89 (0.71–1.11)	0.73 (0.57–0.95)	0.65 (0.47–0.89)	
Yes	0.59 (0.28–1.23)	Ref.	0.68 (0.30–1.53)	0.91 (0.43–1.92)	0.88 (0.40–1.91)	
Physical Activity						0.791
Inactive	0.71 (0.53–0.97)	Ref.	0.82 (0.62–1.10)	0.69 (0.53–0.91)	0.65 (0.44–0.95)	
Active	0.78 (0.54–1.13)	Ref.	0.94 (0.64–1.37)	0.80 (0.54–1.19)	0.68 (0.45–1.04)	
Alcohol Intake						0.217
None	0.66 (0.51–0.84)	Ref.	0.82 (0.63–1.07)	0.69 (0.53–0.90)	0.65 (0.47–0.90)	
Moderate	0.92 (0.33–2.56)	Ref.	1.81 (0.77–4.28)	2.33 (0.93–5.81)	1.55 (0.64–3.77)	
Heavy	1.21 (0.63–2.31)	Ref.	0.93 (0.50–1.72)	0.72 (0.35–1.48)	0.57 (0.28–1.15)	

Adjusted for age + sex + race + education + poverty income ratio + BMI + smoking status + hypertension + diabetes + CKD + depression + physical activity + alcohol intake + seasonal testing + dietary vitamin D.

## Discussion

Using NHANES data, we identified a non-linear association between serum 25(OH)D concentration and *T. gondii* infection. Our cross-sectional analysis demonstrated an inverted U-shaped association, with the highest prevalence of *T. gondii* infection occurring at approximately 51.29 nmol/L of 25(OH)D. The prevalence decreased as 25(OH)D levels deviated from this optimal concentration in either direction. This association remained stable in stratified analyses and in sensitivity analyses.

The literature on the association between serum 25(OH)D levels and *T. gondii* infection is limited and somewhat inconsistent. For example, a study conducted by Kashan et al. in an Iranian population reported a significant increase in the prevalence of *T. gondii* infection in those with serum 25(OH)D deficiency compared to those with normal serum 25(OH)D levels (28.57% vs. 17.14%) (12). Similarly, Rasheed et al. in an observational study of Saudi adult females and observed a similar finding (13). However, both studies shared a common limitation, namely the lack of adjustment for age and other important covariates in the statistical analysis, limiting the generalizability of the findings. In contrast, Kaňková et al. conducted several studies, including two cross-sectional studies (Study A and Study C) and one case–control study (Study B) (14). They did not observe any significant association between serum 25(OH)D concentration and *T. gondii* infection. However, these studies suffered from shortcomings of small sample sizes (Study A: 64 females and 8 males, Study C: 11 males and 18 females) and unrepresentative populations (Study A: mentally retarded population, Study B: Czechs with fertility problems).

Our findings are inconsistent with those of all previous studies. In addition to the previously mentioned limitations, we studied the possible impact of differences in race, dietary habits, and geographic location on the results. The ethnic minorities are at greater risk for serum 25(OH)D deficiency compared with White people (19), which could be attributed to the differences in skin pigmentation, and that non-White people must have increased sun exposure to achieve adequate serum 25(OH)D levels (20). We found that White people had higher 25(OH)D levels, yet despite this, the inverted U-shaped association between serum 25(OH)D and *T. gondii* infection remained unchanged across racial groups in further stratified analyses. This suggests that although ethnicity can influence 25(OH)D levels, it does not alter the association between 25(OH)D and *T. gondii* infection. Moreover, dietary differences can affect serum 25(OH)D levels. The interference of dietary factors in the study was minimized by excluding individuals who took multivitamin agents during the month and adjusted for dietary serum 25(OH)D intake as a covariate to enhance the robustness of the results. Because differences in the geographic location could have influenced inconsistent light exposure times, we adjusted for survey time as a covariate in our analyses. Differences in statistical methods could also lead to inconsistent findings. Previous studies have typically used serum 25(OH)D as a grouping variable, possibly ignoring non-linear relationships. In contrast, we used restricted cubic spline curves to further confirm the non-linear association between serum 25(OH)D and *T. gondii* infection.

Although we confirmed an inverted U-shaped association of serum 25(OH)D with *T. gondii* infection, the exact underlying mechanism remains elusive. This association may be influenced by both the effects of 25(OH)D deficiency and sufficiency on the immune system, as well as the complex immune response to *T. gondii*. Our study identified the highest prevalence of *T. gondii* infection in individuals with 25(OH)D levels below 51.29 nmol/L. According to Holick’s criteria (21), 25(OH)D levels below 50 nmol/L are considered deficient, while levels above 75 nmol/L are deemed adequate. Consequently, the population with 25(OH)D levels below 51.29 nmol/L falls into the deficient category, which may impair immune system function and elevate the prevalence of *T. gondii* infection. 25(OH)D deficiency adversely affects the immune system by reducing the production of antimicrobial peptides like cathelicidin, impairing pathogen clearance, and affecting macrophage phagocytosis and antigen-presenting capacity. It also decreases the number and activity of natural killer (NK) cells, impairs adaptive immune responses, and disrupts T-cell differentiation and function, particularly regulatory T-cell (Treg) production, and antibody production by B-cells, leading to dysregulation of the Th1/Th2 balance (22). Interestingly, the prevalence of *T. gondii* infection was found to increase with higher 25(OH)D levels in the deficient group. This unexpected finding cannot be solely attributed to the negative impact of 25(OH)D deficiency on immune function. It is possible that increased 25(OH)D levels correlate with higher outdoor activity, which in turn raises exposure to *T. gondii*-infected oocysts, thereby increasing infection risk. Approximately 90% of the body’s 25(OH)D is synthesized through skin exposure to sunlight, while the remaining 10% comes from dietary sources, primarily meat (21). Vitamin D deficiency is often observed in individuals with limited sun exposure, inadequate dietary intake, or chronic kidney disease (21). After accounting for dietary vitamin D intake, seasonal variations in sunlight exposure, and the impact of chronic kidney disease on vitamin absorption, we infer that variations in 25(OH)D levels among individuals with concentrations below 51.29 nmol/L are primarily due to sunlight exposure. Our observation supports the hypothesis that increased sunlight exposure may be linked to higher levels of outdoor activity, which could potentially result in greater exposure to *T. gondii* oocysts and an elevated prevalence of infection. Specifically, within the group with 25(OH)D deficiency, physical activity levels appear to increase as 25(OH)D levels rise. Nonetheless, this hypothesis warrants further investigation.

As 25(OH)D levels increase from deficient to adequate, the immune system’s enhancing effects of 25(OH)D become evident. Our study showed a reduced prevalence of *T. gondii* infection when 25(OH)D levels were at or above 51.29 nmol/L, possibly due to 25(OH)D’s specific immunomodulatory effects on *T. gondii* infection. Studies have demonstrated that 1,25(OH)2D3 injections in mice can increase the production of nitric oxide by activating macrophages, subsequently reducing *Toxoplasma* proliferation (23). Further *in vitro* studies have reported that mouse intestinal epithelial cells pretreated with 1,25(OH)2D3 had the lowest number of parasites observed when attacked by *T. gondii* compared with controls, and the inhibitory effect observed was dose-dependent. However, pre-incubation of *T. gondii* with 1,25(OH)2D3 to the cells resulted in no significant difference (11). This suggests that the observed inhibition was due to a 1,25(OH)2D3-mediated cellular effect rather than a direct killing effect on *T. gondii*. This is consistent with the findings of another study that 25(OH)D3 could enhance defense against *T. gondii* by promoting Th2 cell development and modulating cellular immunity. However, these mechanisms linking serum 25(OH)D to *T. gondii* infection remain unproven, and further studies are necessary to elucidate these mechanisms.

Our study had certain limitations. First, we defined *T. gondii* infection based on serum anti-IgG antibody positivity. Although this is a recognized indicator of *T. gondii* infection (4), it could have missed certain acute phase infections (where IgG antibodies were not produced) and individuals with compromised immune responses, potentially limiting the generalizability of our conclusions. Second, causal inferences cannot be drawn owing to the cross-sectional design of this study. Nevertheless, the *in vitro T. gondii* inhibition assay results revealed that high *in vivo* serum 25(OH)D concentrations exert an inhibitory effect on *T. gondii* infection. This hypothesis should be substantiated by further prospective studies. Third, we could not adjust for certain crucial variables, such as a history of cat exposure and raw red meat, due to constraints in the available database. Cats, as the definitive host of *T. gondii*, play a significant role in *T. gondii* transmission. Fourth, *T. gondii* is categorized into three main genotypes (type I, type II, and type III), and the virulence of different genotypic strains of *T. gondii* varies considerably. It remains to be determined whether our conclusions hold across *T. gondii* strains of varying virulence.

Despite these limitations, our study has noteworthy strengths. First, we used a nationally representative sample that facilitated the generalization of our findings to the wider U.S. population. Second, the extensive data collected by NHANES allowed us to control for potential confounding effects stemming from different demographic, socioeconomic, lifestyle, and dietary factors. Third, we validated the robustness of the results across different ethnic and co-morbid patient populations through further stratification and sensitivity analyses.

## Conclusion

In summary, our study revealed an inverted U-shaped association between serum 25(OH)D concentration and *T. gondii* infection. Although the precise molecular mechanisms underlying this connection remain unclear, we believe our research contributes to a deeper understanding of the complex interplay between serum 25(OH)D concentrations and *T. gondii* infection, potentially opening new avenues for designing preventive and therapeutic strategies.

## Data Availability

The datasets presented in this study can be found in online repositories. The names of the repository/repositories and accession number (s) can be found at: https://www.cdc.gov/nchs/nhanes/index.htm.

## Footnotes

^1^ http://www.cdc.gov/nchs/nhanes.html

^2^ https://wwwn.cdc.gov/nchs/nhanes/analyticguidelines.aspx

## References

[1] Attias M Teixeira DE Benchimol M Vommaro RC Crepaldi PH De Souza W. The life-cycle of toxoplasma gondii reviewed using animations. Parasit Vectors. (2020) 13:588. doi: 10.1186/s13071-020-04445-z, PMID: 33228743 PMC7686686

[2] Montoya JG Liesenfeld O. Toxoplasmosis. Lancet (London, England). (2004) 363:1965–76. doi: 10.1016/S0140-6736(04)16412-X 15194258

[3] Tenter AM Heckeroth AR Weiss LM. Toxoplasma gondii: from animals to humans. Int J Parasitol. (2000) 30:1217–58. doi: 10.1016/S0020-7519(00)00124-7, PMID: 11113252 PMC3109627

[4] Jones JL Kruszon-Moran D Elder S Rivera HN Press C Montoya JG. Toxoplasma gondii infection in the United States, 2011-2014. American J Tropical Med Hygiene. (2018) 98:551–7. doi: 10.4269/ajtmh.17-0677, PMID: 29260660 PMC5929212

[5] Mead PS Slutsker L Dietz V McCaig LF Bresee JS Shapiro C. Food-related illness and death in the United States. Emerg Infect Dis. (1999) 5:607–25. doi: 10.3201/eid0505.990502, PMID: 10511517 PMC2627714

[6] Tait ED Hunter CA. Advances in understanding immunity to toxoplasma gondii. Mem Inst Oswaldo Cruz. (2009) 104:201–10. doi: 10.1590/S0074-02762009000200013 19430645

[7] Spellberg B Edwards JE Jr. Type 1/type 2 immunity in infectious diseases. Clinical Infectious Dis: Official Pub Infectious Dis Society of America. (2001) 32:76–102. doi: 10.1086/317537 11118387

[8] Lương KV Nguyễn LT. The role of vitamin D in malaria. J Infect Dev Ctries. (2015) 9:008–19. doi: 10.3855/jidc.3687 25596566

[9] Saad AE Othman AA Ghanem HB Soliman S Alshenawy HA Ghafar MTA. Vitamin D3 supplementation could ameliorate the inflammatory and redox status in the muscular phase of trichinellosis. Parasitol Int. (2023) 94:102737. doi: 10.1016/j.parint.2023.102737, PMID: 36736658

[10] Rajapakse R Mousli M Pfaff AW Uring-Lambert B Marcellin L Bronner C. 1,25-Dihydroxyvitamin D3 induces splenocyte apoptosis and enhances BALB/c mice sensitivity to toxoplasmosis. J Steroid Biochem Mol Biol. (2005) 96:179–85. doi: 10.1016/j.jsbmb.2005.03.002 15939587

[11] Rajapakse R Uring-Lambert B Andarawewa KL Rajapakse RP Abou-Bacar A Marcellin L. 1,25(OH)2D3 inhibits in vitro and in vivo intracellular growth of apicomplexan parasite toxoplasma gondii. J Steroid Biochem Mol Biol. (2007) 103:811–4. doi: 10.1016/j.jsbmb.2006.12.058 17270431

[12] Fakhrieh Kashan Z Shojaee S Keshavarz H Arbabi M Delavari M Salimi M. Vitamin D deficiency and toxoplasma infection. Iran J Public Health. (2019) 48:1184–6. 31341867 PMC6635350

[13] Rasheed Z Shariq A AlQefari GB Alwahbi GS Aljuaythin AI Alsuhibani FS. Toxoplasmosis in immunocompetent Saudi women: correlation with vitamin D. Women's Health (Lond Engl). (2021) 17:174550652110438. doi: 10.1177/17455065211043844 PMC8451252 34541980

[14] Kankova S Bicikova M Macova L Hlavacova J Sykorova K Jandova D. Latent toxoplasmosis and vitamin D concentration in humans: three observational studies. Folia Parasitol. (2021) 68:68. doi: 10.14411/fp.2021.005 33762474

[15] Li B Chen L Hu X Tan T Yang J Bao W. Association of Serum Uric Acid with all-Cause and Cardiovascular Mortality in diabetes. Diabetes Care. (2023) 46:425–33. doi: 10.2337/dc22-1339, PMID: 36490263

[16] KDIGO. 2024 clinical practice guideline for the evaluation and Management of Chronic Kidney Disease. Kidney Int. (2024) 105:S117–s314. doi: 10.1016/j.kint.2023.10.018 38490803

[17] Mao Y Li X Zhu S Geng Y. Association between dietary Fiber intake and risk of depression in patients with or without type 2 diabetes. Front Neurosci. (2022) 16:920845. doi: 10.3389/fnins.2022.920845, PMID: 36389250 PMC9642095

[18] Yalın Sapmaz Ş Şen S Özkan Y Kandemir H. Relationship between toxoplasma gondii seropositivity and depression in children and adolescents. Psychiatry Res. (2019) 278:263–7. doi: 10.1016/j.psychres.2019.06.031, PMID: 31238296

[19] Forrest KY Stuhldreher WL. Prevalence and correlates of vitamin D deficiency in US adults. Nutrition Res (New York, NY). (2011) 31:48–54. doi: 10.1016/j.nutres.2010.12.001 21310306

[20] Clemens TL Adams JS Henderson SL Holick MF. Increased skin pigment reduces the capacity of skin to synthesise vitamin D3. Lancet (London, England). (1982) 319:74–6. doi: 10.1016/S0140-6736(82)90214-8 6119494

[21] Holick MF. Vitamin D deficiency. N Engl J Med. (2007) 357:266–81. doi: 10.1056/NEJMra070553 17634462

[22] Hewison M. Vitamin D and immune function: an overview. Proc Nutr Soc. (2012) 71:50–61. doi: 10.1017/S0029665111001650 21849106

[23] Ghaffarifar F Abdolah Pour M Sharifi Z Dalimi Asl A Al-Kawaz E. The effect of vitamin D3 alone and mixed with IFN-γ on Tachyzoites of toxoplasma gondii (RH strain) proliferation and nitric oxide (NO) production in infected macrophages of BALB/C mice. Iran J Parasitol. (2010) 5:48–56. PMC3279842 22347255

