# Supplementary material for: Inverted U-shaped relationship between serum 25-hydroxyvitamin D concentrations and Toxoplasma gondii infection: a cross-sectional study
Source: Front Public Health. 2024 Aug 29;12:1420932. doi: 10.3389/fpubh.2024.1420932 (PMC11390550; doi:10.3389/fpubh.2024.1420932)
Supplement: Supplementary file 1 [file Table_1.DOCX]

Supplementary Material

# Inverted U-shaped relationship between serum 25-hydroxyvitamin D concentrations and Toxoplasma gondii infection: A cross-sectional study

# Supplementary Figures and Tables

**eTable 1.** The basic characterization of the study population according to *Toxoplasma gondii* infection

|  | *Toxoplasma gondii* IgG antibody | | |  |
| --- | --- | --- | --- | --- |
| variable | Total (n = 10157) | Negative (n =8535) | Positive (n = 1622) | P-value |
| Age, years | 45.38 (0.39) | 44.45 (0.39) | 51.74 (0.72) | < 0.001 |
| Sex, n (%) |  |  |  | 0.002 |
| Female | 5060 (49.73) | 4335 (50.50) | 725 (44.43) |  |
| Male | 5097 (50.27) | 4200 (49.50) | 897 (55.57) |  |
| Race-ethnicity, n (%) |  |  |  | < 0.001 |
| Mexican American | 1514 (8.58) | 1258 (8.28) | 256 (10.63) |  |
| Non-Hispanic Black | 1943 (10.32) | 1627 (10.09) | 316 (11.92) |  |
| Non-Hispanic White | 4614 (68.78) | 4008 (70.16) | 606 (59.33) |  |
| Other Hispanic | 984 (5.51) | 668 (4.48) | 316 (12.55) |  |
| Other Race - Including Multi-Racial | 1102 (6.81) | 974 (6.99) | 128 (5.56) |  |
| Poverty Income Ratio | 2.95 (0.06) | 3.01 (0.06) | 2.59 (0.09) | < 0.001 |
| Education Level, n (%) |  |  |  | < 0.001 |
| Low (<9 years) | 836 (4.35) | 580 (3.46) | 256 (10.47) |  |
| Medium (9–13 years) | 3798 (33.38) | 3100 (32.38) | 698 (40.19) |  |
| High (≥13 years) | 5523 (62.27) | 4855 (64.16) | 668 (49.34) |  |
| BMI, kg/m2 | 28.97 (0.12) | 28.86 (0.12) | 29.66 (0.23) | < 0.001 |
| Smoking status, n (%) |  |  |  | 0.366 |
| Former Smoker | 2305 (23.17) | 1894 (22.88) | 411 (25.16) |  |
| Never Smoker | 5678 (56.62) | 4818 (56.94) | 860 (54.43) |  |
| Current Smoker | 2174 (20.21) | 1823 (20.18) | 351 (20.41) |  |
| Hypertension, n (%) |  |  |  | < 0.001 |
| No | 6233 (65.05) | 5342 (65.80) | 891 (59.95) |  |
| Yes | 3924 (34.95) | 3193 (34.20) | 731 (40.05) |  |
| Diabetes, n (%) |  |  |  | < 0.001 |
| No | 8708 (89.49) | 7383 (89.95) | 1325 (86.29) |  |
| Yes | 1449 (10.51) | 1152 (10.05) | 297 (13.71) |  |
| CKD |  |  |  | < 0.001 |
| No | 8617 (87.66) | 7324 (88.43) | 1293 (82.39) |  |
| Yes | 1540 (12.34) | 1211 (11.57) | 329 (17.61) |  |
| Depression, n (%) |  |  |  | 0.977 |
| No | 9194 (91.83) | 7735 (91.83) | 1459 (91.81) |  |
| Yes | 963 (8.17) | 800 (8.17) | 163 (8.19) |  |
| Physical Activity, n (%) |  |  |  | < 0.001 |
| Inactive | 4996 (44.14) | 4067 (42.99) | 929 (51.98) |  |
| Active | 5161 (55.86) | 4468 (57.01) | 693 (48.02) |  |
| Alcohol Intake, n (%) |  |  |  | < 0.001 |
| None | 7711 (72.25) | 6404 (71.27) | 1307 (78.94) |  |
| Moderate | 1735 (20.53) | 1535 (21.58) | 200 (13.36) |  |
| Heavy | 711 (7.23) | 596 (7.16) | 115 (7.70) |  |
| Seasonal Testing, n (%) |  |  |  | 0.204 |
| May 1 through October 31 | 5267 (56.11) | 4442 (56.45) | 825 (53.84) |  |
| November 1 through April 30 | 4890 (43.89) | 4093 (43.55) | 797 (46.16) |  |
| Dietary vitamin D, (mg) | 4.81 (0.08) | 4.79 (0.09) | 4.89 (0.17) | 0.584 |

**Notes:** Data are represented as the weighted proportion (%) or mean ± SE.

**Abbreviations:** BMI, body mass index; CVD, cardiovascular disease

**eTable 2.** Sensitivity Analysis: ORs and 95% CIs of *Toxoplasma gondii* infection according to serum 25(OH)D levels after Utilizing Surveyimpute for Multiple Interpolation (n=7439)

| Variable | Total | T. gondii-seropositive | Non-adjusted Model | p Value |
| --- | --- | --- | --- | --- |
|  | n | n (%) | HR (95% CI) |  |
| Serum 25(OH)D levels (nmol/L) | 17596 | 2245 (12.8) |  |  |
| Quintile 1(<41.45) | 3518 | 447 (12.7) | 0.95 (0.82–1.11) | 0.517 |
| Quintile 2 (41.46-54.74) | 3518 | 446 (12.7) | Ref. |  |
| Quintile 3 (54.75-66.04) | 3512 | 470 (13.4) | 1.05 (0.90–1.21) | 0.558 |
| Quintile 4 (66.05-81.05) | 3528 | 430 (12.2) | 0.94 (0.81–1.1) | 0.428 |
| Quintile 5 (>81.06) | 3520 | 452 (12.8) | 0.84 (0.72–0.98) | 0.030 |

**Notes:** adjusted for age+sex+race+education+poverty income ratio+BMI+smoking status+hypertension+diabetes+CKD+depression+physical activity+alcohol intake+seasonal testing+ dietary vitamin D
